# Transforming Epilepsy Care: A Comprehensive Review of Technological Innovations, Artificial Intelligence Advancements, and Precision Tools for Enhanced Seizure Management and Patient Outcomes

**DOI:** 10.1155/sci5/5915062

**Published:** 2026-01-29

**Authors:** Mukul Shyam, Naincy Kaushal, Manish Yadav, Prathap Srirangan, Sabina Evan Prince

**Affiliations:** ^1^ Department of Biotechnology, School of Biosciences and Technology, VIT University, Vellore, Tamil Nadu, India, vit.ac.in; ^2^ Structural Biology, Biophysics and Biochemistry Lab, CSIR-Institute of Microbial Technology, Chandigarh, India, csir.res.in; ^3^ Department of Chemistry, Central Ayurveda Research Institute, Jhansi, India

**Keywords:** artificial intelligence, electroencephalography, epilepsy, medical devices, neuromodulation

## Abstract

Epilepsy is a neurological disorder characterized by repeated, unprovoked seizures arising from abnormal electrical activity in the brain. The management of epilepsy presents several significant challenges. One of these challenges involves drug resistance, where seizures persist despite treatment with multiple antiepileptic drugs, requiring a tailored approach often involving complex medication regimens or alternative therapies. Additionally, antiepileptic medications can cause a range of side effects, from minor discomfort to severe complications, which can affect patient adherence and overall well‐being. Accurate classification and diagnosis of seizures are crucial yet complex, potentially resulting in misdiagnosis and inappropriate treatments. These challenges can be addressed through the utilization of medical devices. A comprehensive assessment of medical apparatuses is utilized in the management of epilepsy, focusing on both implanted and non‐invasive technologies. This examination encompasses implanted devices, such as neuromodulators, which are surgically inserted to address seizures in individuals with epilepsy who are resistant to pharmaceutical treatment, as well as noninvasive devices, including wearable technologies and mobile applications. These noninvasive devices enhance seizure detection and monitoring by leveraging advancements in electroencephalography, allowing for real‐time data collection. Consequently, there has been an improvement in diagnostic precision and the facilitation of tailored treatment approaches.

## 1. Introduction

Epilepsy is a chronic neurological disorder characterized by recurrent seizures due to abnormal electrical activity in the brain [1]. The pathogenesis of epilepsy involves complex mechanisms, including hypersynchronous discharge in the epileptogenic zone and alterations in brain network dynamics, leading to increased seizure susceptibility. Epilepsy affects an estimated fifty million individuals globally without considering sociodemographic factors. Research has shown that the point prevalence of epilepsy ranges from 4 to 10 per 1000 individuals, establishing it as one of the most common neurological disorders. The estimated incidence rate of epilepsy is 50–60 per 100,000 person‐years, and a considerable proportion of individuals, up to 8%, experience at least one seizure during their lifetime [2, 3]. Diagnosing epilepsy poses a significant challenge due to the restricted availability of diagnostic modalities such as electroencephalography (EEG), magnetic resonance imaging, computed tomography, and supplementary tests such as lumbar puncture and genetic testing. The treatment of epilepsy presents a significant challenge not only in terms of diagnosis but also because of the adverse effects associated with antiepileptic drugs (AEDs), which include cognitive impairment, nausea, Stevens‒Johnson syndrome, behavioral changes, and osteopenia. Moreover, these medications are known to induce hepatotoxicity, pancreatitis, leukopenia, and thrombocytopenia and exhibit teratogenic effects when administered during pregnancy. AED resistance, a condition affecting approximately one‐third of individuals with epilepsy, is characterized by seizures persisting despite adequate trials of two or more AEDs. For individuals suffering from drug‐resistant epilepsy, there are various alternatives available beyond conventional AEDs. Implantable devices provide supplementary options; for example, for vagus nerve stimulation, electrical impulses are administered to the vagus nerve to alleviate the occurrence of seizures [4]. Moreover, responsive neurostimulation supervises brain activity, and stimulation is administered when seizures are anticipated. The technique of deep brain stimulation (DBS) entails the insertion of electrodes in specific brain areas to regulate neural activity [5]. Non‐invasive devices such as transcranial magnetic stimulation (TMS) utilize magnetic fields to provoke brain activity, potentially lessening seizures. Moreover, non‐invasive devices such as TMS, accelerometers, and wearable devices apply magnetic fields to increase brain function, reducing seizures. Novel technologies such as optogenetics, focused ultrasound, and artificial intelligence/machine learning operate on fundamental principles and contribute to the management and identification of seizures through EEG data, although they necessitate comprehensive validation and the resolution of challenges concerning safety, availability, and clinical confirmation.

## 2. Implantable Devices

Neuromodulation can be described as using electrical or magnetic stimuli to modify nerve activity, particularly to address neurological disorders such as epilepsy. The main goal of employing neuromodulation techniques for epilepsy is to hinder or reduce the frequency and severity of seizures by precisely targeting specific brain regions or pathways [6]. Implantable devices used as neuromodulators are specialized medical devices designed to modulate or alter the activity of the nervous system through electrical stimulation or drug delivery. In the domain of epilepsy, implantable devices are surgically implanted into the body to assist in the management of seizures and improve the quality of life for patients with drug‐resistant epilepsy. These devices commonly deliver electrical stimulation to nerves or specific areas of the brain that play a role in initiating or modifying seizures [7]. The primary types of implantable devices employed in epilepsy are explained below.

### 2.1. Vagus Nerve Stimulator (VNS)

The vagus nerve is a crucial component of the autonomic nervous system that significantly influences various bodily functions. The vagus nerve, after originating from the medulla oblongata, exits the cranium through the jugular foramen and travels down the neck within the carotid sheath along the common carotid artery and the internal jugular vein. Compared with the other cranial nerves, the vagus nerve connects many visceral organs with the brainstem and the cortex, given its widespread course and distribution through the autonomic nervous system interface [8]. The evolution of VNS therapy advanced through initial experimental investigations in animals, which revealed encouraging outcomes in reducing the frequency of seizures. The debut of the initial implantable VNS device was authorized by the U.S. Food and Drug Administration (FDA) in 1997 to manage epilepsy in individuals aged 12 years and older.

VNS, an open‐loop device, administers scheduled electrical stimulation to the vagus nerve, typically for durations of 30–60 s at regular intervals. The intensity of the current is adjusted gradually to a minimum of 1.50 mA within 10–12 weeks postimplantation, depending on the patient’s tolerance levels. Additionally, a wrist magnet is provided to the patient to activate the pulse generator and administer supplementary stimulation. VNS functions are based on the premise that mild electrical stimulation of the vagus nerve can interfere with or regulate abnormal brain activity, leading to seizures [9]. The VNS device is typically surgically inserted beneath the skin in the thoracic region, with a connecting lead wire linking it to the left vagus nerve in the cervical area. Owing to efferent projections to the sinoatrial node, the left vagus nerve is preferred for stimulation.

The antiseizure effects of VNS are facilitated by the modulation of neurotransmitter release (e.g., GABA, glutamate) and changes in synchronization within thalamocortical networks, encompassing phase‐amplitude coupling across various frequency bands [10]. This modulation facilitates cortical desynchronization and neuroplasticity, with activation of noradrenergic *α*2 receptors and astrocytic calcium signaling playing a role in long‐term effects [11].

VNS results in a variety of negative outcomes, as elucidated in the literature. Common adverse events include alterations in voice quality, tingling sensations, coughing, breathlessness, and discomfort. Moreover, laryngeal complications such as changes in voice characteristics and partial or complete loss of voice function have been observed, which are frequently linked to issues with the device, such as high resistance and incorrect frequency allocation. In exceptional instances, uncontrollable episodes of hiccups have been recorded post‐VNS insertion, leading to serious repercussions such as Mallory–Wiss syndrome and the necessity for intensive medical attention [12].

### 2.2. Responsive Neurostimulation System (RNS)

RNS has emerged as a valuable treatment option for patients with drug‐resistant epilepsy, particularly those with focal epilepsy or multifocal epilepsy who are ineligible for surgical intervention. The NeuroPace RNS system not only provides safe and well‐tolerated stimulation but also collects crucial long‐term electrocorticographic data, aiding in seizure risk assessment, treatment response evaluation, and future surgical planning [13]. The RNS system, developed by NeuroPace, possesses a significant historical background that originated in the late 1990s with the emergence of the idea of utilizing targeted electrical stimulation to regulate brain activity for the treatment of epilepsy. In 2013, the FDA approved the RNS system, allowing its utilization for clinical purposes in the United States.

Electrocorticography (ECoG) data, in which subdural grids and stereotaxic depth electrodes are placed on the cortex for weeks at a time to elucidate seizure origination, are commonly obtained during drug‐resistant epilepsy workups. RNS was the first to use recorded ECoG data to direct the delivery of electrical stimulation to control seizures [14, 15]. In addition to its primary role in responsive neurostimulation, the RNS system archives and retains ECoG data for medical assessment, aiding in the accessibility and examination of long‐term ECoG data. ECoG data include identifying epileptiform activity, responsive stimulation, extended detection events, patient‐initiated magnet swipes, saturation resulting from high‐amplitude activity, and detecting disturbances such as 60 Hz line interference.

The RNS apparatus comprises a neurostimulator connected to two leads, each of which may be either NeuroPace depth leads or NeuroPace cortical strip leads. Both leads are equipped with four electrodes at the distal end, placed at the location of the seizure onset zone or seizure spread (more recently), and four electrodes at the proximal end are attached to the neurostimulator [14, 16]. Information from the RNS device can be collected, and adjustments can be made via the programmer, remote monitor, and patient data management system (PDMS) database [17]. The programmer enables the physician to modify stimulation parameters, whereas the remote monitor serves as a home‐monitoring tool for patients to upload ECoG data recorded by the RNS device to the PDMS database, a cloud storage system for ECoG data, for later review by the physician. Following implantation, the RNS device is initially programmed to passively record ECoG data without administering electrical stimulation for a period required to define seizure neurophysiology, referred to as the programming epoch. The stimulation parameters are subsequently configured into the RNS device, enabling it to administer electrical stimuli upon detecting epileptiform activity. The recommended initial responsive therapy settings are as follows: frequency: 200 Hz; burst duration: 100 ms; current: adjusted to achieve a charge density = 0.5 μC/cm^2^; pulse width: 160 μs. The primary parameter adjusted when altering the stimulation settings is the charge density, which is increased by 0.5 μC/cm^2^ at each programming session if the response at the current charge density level is deemed unsatisfactory. The key distinction between RNS and other neuromodulatory devices lies in the fact that RNS operates as a closed‐loop system with sensing capabilities. This finding indicates that the implanted neurostimulator provides direct electrical stimulation in response to the identification of specific patterns of electrographic activity previously identified by the physician as epileptiform activity [18].

Fifty individuals, with a median age of 39.5 years and 64% female, were subjected to treatment with RNS for drug‐resistant epilepsy at Emory University School of Medicine between 2005 and 2020. Among them, 37 participants diligently recorded their seizure occurrence both pre‐ and post‐implantation. The collected data demonstrated an 88% median decrease in seizure frequency after 6 months, with a response rate of 78% (defined as a reduction of 50% or more in seizure frequency), and 32% of the subjects experienced freedom from severe seizures during this timeframe. Evaluations did not reveal any statistically notable differences in cognitive, psychiatric, or quality‐of‐life results at 6‐ and 12‐month post‐implantation compared with the baseline values before the procedure, irrespective of the observed seizure outcomes [19].

RNS is a valuable therapeutic approach for epilepsy; however, it is accompanied by certain inherent risks. The surgical installation of the RNS device may result in complications such as infections, hemorrhage, or damage to adjacent brain structures. Moreover, device‐related issues such as malfunction or the necessity for reprogramming can manifest. The neurological side effects, including tingling, muscle spasms, or alterations in sensory perception, can be attributed to the stimulation process. Furthermore, cognitive implications such as memory deficits, concentration difficulties, and mood alterations have been documented.

### 2.3. DBS

DBS, which is primarily utilized in the management of movement disorders such as Parkinson’s disease, is now being employed as a therapeutic approach for individuals suffering from drug‐resistant epilepsy [20]. The potential of DBS as a treatment option for epilepsy has been under investigation, especially for patients who exhibit poor responses to traditional medications or alternative surgical procedures. The history of employing DBS in the treatment of epilepsy traces back to initial experiments and clinical trials conducted in the early 2000s.

Recent progress in the use of DBS for epilepsy has underscored the importance of factors such as electrode placement, stimulation parameters, and the specific type of epilepsy in influencing clinical results. Situated in the anterior region of the thalamus, the anterior nucleus of the thalamus (ANT) is a nuclear complex with strong connections to the hippocampal formation, cingulate cortices, and inferior parietal lobule, playing crucial roles in limbic and temporal lobe epilepsies as part of the extended hippocampal formation. ANT‐DBS has exhibited efficacy in the management of focal and secondarily generalized forms of DRE [21, 22]. Positioned within the caudal intralaminar group of thalamic nuclei, the centromedian nucleus (CM) displays a broad connectivity pattern, including projections to the striatum, thalamic nuclei, reticular formations, and somatomotor cortices, representing a typical example of the cortical–thalamic–cortical system. CM‐DBS is particularly successful in addressing generalized epilepsies and patients diagnosed with Lennox–Gastaut syndrome, which is beneficial for individuals with primary generalized DRE [23].

DBS relies heavily on the precise selection of its stimulation parameters, including voltage, frequency, pulse width, and stimulation mode. The optimal stimulation parameters are those that effectively reduce seizures with minimal side effects and device battery consumption. In patients with intractable epilepsy (IE), DBS is typically initiated with voltage settings of 5–6 at frequencies between 130 and 180 Hz and pulse widths ranging from 60 to 90 μs in monopolar intermittent stimulation mode (1 min ON and 5 min OFF). High‐frequency stimulation has shown efficacy in reducing seizures, whereas low‐frequency stimulation is generally inefficacious and may even worsen existing seizures. The primary goal of selecting stimulation parameters for successful DBS is to induce EEG desynchronization while decreasing or potentially eliminating interictal epileptiform discharges. In the context of Parkinson’s disease, another neurological condition characterized by abnormal neuronal synchronization, the standard frequency of subthalamic nucleus stimulation ranges from 130 to 180 Hz. A study indicated that frequencies at or above 90 Hz produced optimal outcomes, whereas frequencies at 10 or 50 Hz failed to achieve the desired clinical effects [24].

## 3. Non‐Invasive Devices

Imaging tools such as ultrasound, MRI, and CT provide intricate visualizations of internal structures, aiding in the identification and characterization of illnesses without the necessity of surgical intervention. Monitoring equipment such as ECGs, pulse oximeters, and continuous glucose monitors measures essential physiological parameters over a period, enabling immediate evaluation of health conditions and disease advancement [25]. Non‐invasive devices for epilepsy, such as optically pumped magnetometers (OPMs) and non‐invasive mobile EEG solutions, represent promising advancements in the realm of epilepsy diagnosis and management. The utilization of OPMs positioned directly on the scalp serves to increase the signal‐to‐noise ratio, thereby improving sensitivity toward deep sources and facilitating the recording of seizures with heightened levels of patient‐friendliness and cost‐effectiveness [26, 27]. Conversely, non‐invasive mobile EEG solutions offer a convenient and dependable approach to monitoring patients with chronic conditions, demonstrating positive feedback from both patients and healthcare providers, satisfactory signal integrity, and ongoing research endeavors aimed at enhancing the accuracy of seizure detection.

### 3.1. Accelerometers

**Figure 1:**
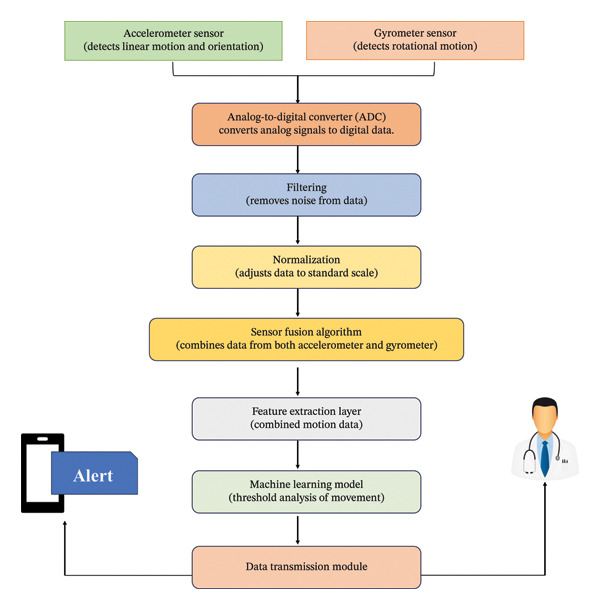
The depiction delineates a framework for the detection and examination of human motion. Commencing with accelerometer and gyroscope sensors that record linear and rotational motion data, respectively. This unprocessed data goes through a sequence of procedures, encompassing analog‐to‐digital conversion, filtration, standardization, and sensor integration to amalgamate data from both sensors. The refined data are then input into a layer for feature extraction, followed by a machine learning model that scrutinizes the motion patterns. The framework can issue an alert, potentially indicating a particular motion or activity, and the data are dispatched for further medical assessment.

Accelerometers are essential in the management of epilepsy, as they facilitate the identification of seizures. These devices are sensors designed to measure acceleration, which is defined as the rate of change over time [28, 29]. The utilization of accelerometers in wearable devices for epilepsy management relies on microelectromechanical system technology [30]. By detecting alterations in capacitance or producing electrical charges when in motion, these sensors gauge acceleration. They typically assess acceleration across three dimensions (x‐, y‐, and *z*‐axes) and are proficient in recognizing specific movements related to seizures, such as convulsions or shaking. Embedded algorithms in wearable devices analyze accelerometer data instantly to differentiate seizure activity from ordinary movements, consequently alerting caregivers or healthcare providers, as shown in Figure 1. This continuous monitoring capacity allows for thorough monitoring of seizure frequency and intensity over prolonged durations, significantly contributing to treatment planning and assessment. Studies have proven that the use of wrist‐worn accelerometers can accurately identify epileptic seizures with minimal false alarms, as evidenced by research that achieved a seizure detection accuracy of 95.7% and a low false alarm rate of 14.8% through a hidden Markov model technique [31]. Although accelerometers are advantageous in identifying convulsive seizures in epilepsy management, they face various constraints affecting their overall efficiency. Principally, these sensors excel in capturing movements linked to tonic‐clonic seizures but may encounter challenges in spotting nonconvulsive seizures such as absence seizures, which lack substantial motor activity, as shown in Figure 1.

Algorithms deciphering accelerometer data may sometimes yield incorrect positives or negatives, erroneously categorizing normal movements as seizures or missing actual seizure occurrences. Accelerometer reliance on movement presents difficulty in detecting seizures during periods of minimal activity, such as nocturnal seizures.

### 3.2. Empatica Embrace2

Embrace2, developed by Empatica, represents the most recent advancement in epilepsy sensors from the company, which has solidified its position as a prominent authority in the creation of devices tailored for seizure detection. In Europe, Embrace2 has obtained official recognition as a medical instrument, and it has also received approval from the FDA in the United States. This wearable sensor, which is designed to be worn on the wrist, uses electrodermal activity measurements to identify alterations in specific skin attributes, such as sweat secretion, a commonly observed sign of potential seizure episodes [32]. Equipped with an accelerometer, gyroscope, and temperature sensor, Embrace2 is capable of tracking repetitive movements and variations in body heat, features that significantly enhance its ability to recognize possible tonic‐clonic seizures.

### 3.3. Ambulatory EEG

Ambulatory EEG, also known as Amb EEG, is a technique developed for the extensive monitoring of cerebral activity in natural environments beyond the confines of conventional clinical settings. This approach uses a mobile recording device equipped with scalp electrodes, which allows individuals to wear the equipment comfortably for extended periods, typically lasting anywhere from 24 to 72 h or even longer. By adopting this strategy, a continuous stream of EEG data is captured during various routine activities, including sleep, work, and physical exercise, thereby offering valuable insights that may elude detection in brief clinical EEG sessions. The application of ambulatory EEG is particularly advantageous in the diagnostic process of epilepsy and other neurological disorders characterized by sporadic symptoms or infrequent episodes. By providing real‐time data acquisition and visualization on mobile devices, mobile EEG systems enhance the diagnostic capabilities for epilepsy, enabling personalized and efficient treatment decisions based on the patient’s specific needs and condition [33].

Ambulatory EEG incorporates a variety of specialized systems designed for distinct diagnostic and research purposes. With modern technology, wireless ambulatory EEG systems enable real‐time data transmission, allowing for remote monitoring and immediate analysis via cloud‐based platforms or external devices. Dry electrode ambulatory EEG devices eliminate the requirement for conductive gel, providing convenience and comfort while maintaining signal quality. Mobile application‐based ambulatory EEG integrates smartphone or tablet interfaces for seamless data collection and patient engagement [34, 35]. Integration of EEG and video monitoring systems enables simultaneous recording of brain activity and visual documentation of clinical occurrences, thereby facilitating comprehensive diagnostics and treatment assessment.

### 3.4. Subscalp EEG

Subscalp EEG solutions, such as EpiMinder’s Minder and UNEEG Medical’s 24/7 EEG SubQ, represent a significant advancement in long‐term seizure monitoring [36]. These minimally invasive technologies involve inserting small electrodes beneath the scalp, enabling continuous EEG recording for weeks, months, or even years, while allowing patients to carry out their daily activities [37]. EpiMinder’s Minder provides signal quality comparable to a standard 10–20 scalp EEG, with less noise and better patient mobility, and has been effective over multiple years. UNEEG’s 24/7 EEG SubQ uses a small subcutaneous implant combined with an external device to capture and transmit EEG data, with research showing it can operate for over 30 days of continuous home monitoring [38]. A home‐use study recorded 490 days of EEG data, detecting 338 electrographic seizures and revealing substantial underreporting in patient seizure diaries, with no serious adverse effects observed [39]. Comparative studies show that subscalp EEG signals closely resemble scalp EEG in morphology and timing when electrodes are correctly placed, but with improved stability and fewer artifacts. Unlike traditional surface EEG, which offers excellent temporal resolution but limited spatial sensitivity and is prone to artifacts from muscle activity, eye movements, and electrical noise, subscalp systems maintain higher fidelity over longer periods and can detect electrographic seizures that surface recordings may miss, especially those from deep or subtle cortical sources [40]. The combination of durability, accuracy, and minimal invasiveness makes subscalp EEG an increasingly valuable tool for objectively assessing seizure burden, guiding therapy adjustments, and predicting seizures in everyday environments.

### 3.5. Ear EEG

In‐ear EEG systems are an innovative type of unobtrusive, wearable neurotechnology designed to provide extended ambulatory seizure monitoring while minimizing the appearance and discomfort associated with traditional scalp EEG [41]. These devices typically use custom‐fitted earpieces or soft electrodes placed within the ear canal to record brain activity from regions near the temporal lobe, a common seizure zone in focal epilepsy [42]. Their discreet design offers a significant advantage for social acceptability, reducing the stigma usually linked to visible scalp electrodes, and they can be worn comfortably during everyday activities without drawing attention [43]. Research has demonstrated promising results: in a clinical feasibility study with patients suffering from refractory focal epilepsy, in‐ear EEG detected about 86% of seizures recorded by intracranial or scalp EEG, with a low false alarm rate of roughly 0.1 per day. Sensitivity was notably higher for temporal lobe seizures because of the proximity of the recording site to the seizure focus. Morphological and spectral analyses show a strong correlation between in‐ear and scalp EEG signals during both ictal and interictal events, especially in temporal regions, indicating their potential as a reliable substitute for short‐ or long‐term monitoring [44]. Additionally, incorporating these systems into common wearable devices, such as advanced earbuds or hearing aids, offers a way to achieve continuous, discreet, and socially integrated brain monitoring.

### 3.6. TMS

TMS is a nascent modality employed in the management of epilepsy. This pioneering method involves the application of magnetic fields to provoke neuronal activity in the brain. Approval from the FDA for the treatment of major depressive disorder was granted to TMS in 2008, signifying a notable milestone in acknowledging TMS as a viable therapeutic option for patients with treatment‐resistant depression. The duration of TMS sessions may vary depending on the specific protocol utilized and the condition under treatment. Typically, a single TMS session lasts between 20 and 40 min [45]. Nevertheless, the total duration and frequency of sessions may differ based on the treatment plan recommended by the healthcare provider. TMS has promising efficacy in the treatment of epilepsy. Two primary modalities are utilized: repetitive TMS (rTMS), which is characterized by the repetitive administration of multiple pulses, and single‐pulse TMS (sTMS), which involves the delivery of a solitary pulse during each session. In epilepsy research, TMS serves two main purposes: delineating the epileptogenic zone within the brain to aid in surgical planning and potentially modulating the epileptic activity itself by stimulating or inhibiting the seizure focus. The mechanism of action of TMS revolves around the creation of magnetic fields that induce electrical currents in cerebral tissue. Low‐frequency (≤ 1 Hz) TMS over the epileptogenic cortex has demonstrated a reduction in cortical excitability and seizure frequency. A study examining seven randomized controlled trials reported a mean seizure reduction of approximately 30%–40% over several weeks, especially in neocortical focal epilepsy. The most compelling data pertains to focal motor seizures and cortical dysplasia; however, the results are frequently temporary and necessitate maintenance sessions [46, 47]. High‐frequency TMS, conversely, may intensify epileptiform activity and is typically contraindicated in epilepsy treatment [48].

### 3.7. Transcranial Direct Current Stimulation (tDCS)

**Figure 2:**
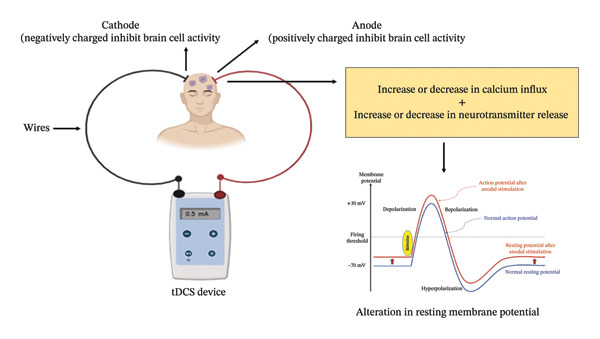
The illustration depicts the fundamental principles of transcranial direct current stimulation (tDCS). Through the utilization of electrodes positioned on the scalp, a tDCS apparatus administers a feeble electrical current to the brain. By modifying the influx of calcium ions, the cathode (negative electrode) and anode (positive electrode) have an impact on brain cell activity, thereby influencing the release of neurotransmitters. This adjustment in neuronal activity has the potential to induce alterations in the resting membrane potential and consequently affect brain functionality.

tDCS is a form of non‐invasive neuromodulation that delivers a weak electric current to the brain via electrodes placed on the scalp, generating a subtle electric field that influences cortical neuron polarization. Research findings have suggested that tDCS shows promise in enhancing cognitive functions, particularly numerical skills, by modulating cortical excitability through synaptic mechanisms [49]. Anodal stimulation is thought to increase cortical excitability, whereas cathodal stimulation is believed to decrease it. The aim of tDCS in epilepsy‐related regions is to potentially increase the seizure threshold or disrupt abnormal neuronal synchronization, a characteristic feature of epilepsy, as mentioned in Figure 2.

One study investigated the effects of tDCS on reducing epileptiform activity and seizure frequency in individuals with drug‐resistant focal epilepsy stemming from cortical malformations [50]. This double‐blind, randomized, sham‐controlled trial involved applying cathodal tDCS, which reduces excitability, for 20 min with a current intensity of 1 mA over the suspected epileptogenic area. Following actual tDCS treatment, there was a significant decrease in the occurrence of epileptiform discharges. Another study, a 2020 systematic study, indicated that approximately 50% of patients saw a reduction of at least 50% in seizures in the short term, accompanied by minimal adverse effects, primarily scalp tingling and slight erythema. tDCS has demonstrated potential as an additional treatment for drug‐resistant epilepsy in juvenile patients [51].

Regarding practical integration, both TMS and tDCS are presently constrained by inconsistent study procedures, brief follow‐up periods, and an absence of extensive phase III trials. Neither modality possesses regulatory approval for the treatment of epilepsy in most countries and is mostly provided in research or specialized neurostimulation facilities. Practical obstacles encompass the identification of appropriate stimulation targets using neuroimaging/EEG, the customization of protocols, and the establishment of reimbursement mechanisms [52, 53].

## 4. Comparative Summary of Implantable vs. Wearable EEG Systems

Implantable EEG devices (e.g., EpiMinder, UNEEG 24/7 EEG SubQ, NeuroPace RNS) offer high‐fidelity, long‐term recordings that are impervious to skin or movement aberrations, facilitating the dependable identification of both clinical and subclinical seizures—especially focal events—over extended periods, ranging from weeks to years. They necessitate minimally to moderately invasive insertion and have obtained CE marking in Europe, along with restricted FDA approval in the United States. Conversely, wearable EEG systems (e.g., Epitel, mBrainTrain) are non‐invasive, portable, and well‐accepted, making them appropriate for short‐ to medium‐term monitoring in outpatient or home environments. Although they provide enhanced ease and expedited deployment, they are more prone to signal loss and exhibit diminished spatial coverage, rendering them more suitable for generalized seizure detection. The majority are adjunctive diagnostic instruments, with many still in research or preliminary clinical application, as detailed in Table 1.

**Table 1:** Comparative features of implantable versus wearable EEG systems for epilepsy monitoring.

Feature	Implantable EEG systems	Wearable EEG/seizure detection devices	References
Invasiveness	Minimally invasive subcutaneous implantation requires a surgical procedure	Non‐invasive; worn externally on the wrist, scalp, or behind the ear	[54, 55]
Monitoring duration	Continuous long‐term monitoring (months–years)	Short‐ to medium‐term (hours–weeks), depending on battery life and comfort	[56, 57]
Recording fidelity	High stability, low motion artifacts; captures focal and subclinical seizures reliably	Variable; prone to noise/motion artifacts; sensitivity highest for generalized tonic‐clonic seizures	[58]
Seizure types detected	Focal, generalized, subclinical seizures	Primarily generalized motor seizures; limited detection for focal/non‐motor events	[59, 60]
Regulatory status	Minder®—FDA De Novo authorization (2025), CE mark in EU	Empatica Embrace2—FDA cleared for GTCS detection; CE marked; others under clinical investigation	[61]
Primary clinical use	Chronic seizure burden tracking; optimizing medication/surgery candidacy	Ambulatory seizure alerting; adjunct to seizure diaries; rapid EEG triage in acute care	[62]

## 5. Intraventricular and Convection‐Enhanced Delivery (CED) System

Intraventricular (ICV) delivery entails the implantation of a catheter into the lateral ventricles, which is linked to an infusion pump for the direct administration of anticonvulsant drugs into the cerebrospinal fluid (CSF). This approach circumvents the blood–brain barrier, resulting in significantly elevated drug concentrations at seizure foci while maintaining little systemic exposure. In preclinical rat kindling models, continuous intraventricular infusion of valproate successfully suppresses seizures while circumventing the ataxia and drowsiness associated with systemic treatment, resulting in increased brain concentrations without increasing plasma or liver levels [63]. A groundbreaking study in humans utilizing a long‐term implanted ICV delivery system for valproic acid in drug‐resistant temporal lobe epilepsy revealed elevated CSF levels (mean 45 μg/mL), diminished serum levels (4–14 μg/mL), favorable tolerability, and substantial seizure reduction, with two subjects attaining extended seizure freedom and enduring benefits for up to 2.5 years. A long‐term follow‐up of a group demonstrated sustained efficacy, with responder rates of approximately 82% at 24 months, and no significant safety issues were identified [64].

CED utilizes stereotactic insertion of one or more brain catheters linked to a pump, creating a pressure gradient that facilitates the bulk flow of therapeutic agents into the brain interstitium, enabling deeper and more uniform tissue distribution compared to diffusion‐based techniques [65]. Instances of antiseizure agents administered using these techniques include, for example, valproate administered intraventricularly through continuous infusion, which provides robust anticonvulsant effects in both animal models and human studies, achieving elevated brain concentrations with low systemic exposure. Gabapentin and adenosine: Although less extensively researched clinically, these substances have been delivered via ICV administration in animal models—gabapentin via osmotic pumps for targeted delivery and adenosine via polymer implants, both exhibiting seizure control with minimal peripheral exposure [63, 66, 67].

### 5.1. Other Targeted Infusion Systems for Epilepsy

Alongside ICV and CED, various other targeted infusion methodologies are under investigation to boost local medication bioavailability in focal epilepsies. Intrathecal drug delivery entails the infusion of anticonvulsant drugs into the lumbar subarachnoid space by an implanted pump (e.g., Medtronic SynchroMed II), enabling CSF circulation to transport agents to the brain [68]. Although mostly utilized for spasticity therapy, intrathecal administration of baclofen and anesthetic drugs has been tested in refractory status epilepticus, resulting in seizure suppression in otherwise intractable instances [69]. For example, subpial and subdural infusion systems place catheters directly on the cortical surface above epileptogenic zones, facilitating exceptionally high local concentrations without entering the ventricles. In experimental models of temporal lobe epilepsy, subpial infusion of GABA_A agonists like muscimol has produced significant, localized seizure suppression [70]. Where local infusions facilitated by nanoparticles and liposomes provide extended medication release and less washout. Liposomal formulations of valproate and carbamazepine administered through CED in experimental epilepsy sustained therapeutic concentrations in seizure foci for prolonged durations, reducing the necessity for repeated infusions [71, 72].

## 6. Emerging Technologies in Epilepsy

Emerging technologies, including optogenetics, focused ultrasound, and machine learning/AI, are significantly transforming the treatment of epilepsy. Optogenetics facilitates the precise regulation of neuronal activity through the utilization of light, potentially enabling targeted inhibition or activation of specific brain regions implicated in seizure initiation [73]. Focused ultrasound is a noninvasive technique for either ablating or modulating brain tissue, offering a promising alternative to conventional surgical procedures. In parallel, machine learning and AI are revolutionizing the management of epilepsy through the analysis of intricate patterns of brain activity to forecast the onset of seizures, optimize parameters for neurostimulation, and customize treatment approaches on an individualized level [74]. These advancements collectively hold the potential for more efficacious and personalized strategies in the treatment of epilepsy.

### 6.1. Optogenetics

**Figure 3:**
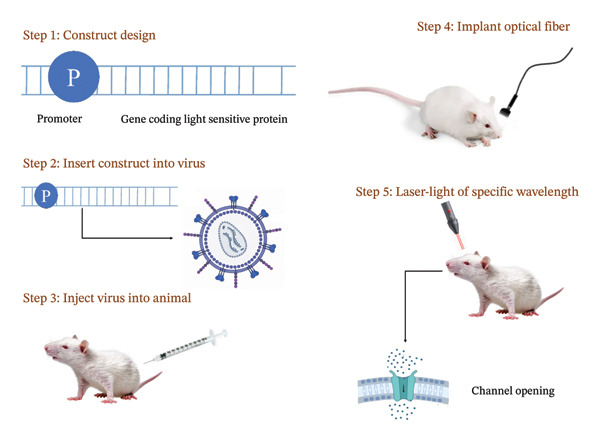
The illustration depicts the procedure of optogenetics, commencing with the assembly of a genetic sequence that includes a promoter and a gene encoding a light‐responsive protein. Subsequently, this genetic construct is introduced into a virus, which is then administered to an animal. Afterward, an optical fiber is surgically implanted into the brain of the animal. Ultimately, a laser beam with a specific wavelength is transmitted through the optical fiber to stimulate the light‐sensitive protein, resulting in the activation of ion channels in the specified neurons.

Optogenetics represents an innovative methodology that combines light and genetics to intricately regulate cellular functions in organisms with exceptional spatiotemporal precision and minimal invasiveness. Through the introduction of natural or synthetic photoreceptors into cells, optogenetics facilitates the manipulation of light‐insensitive cells, rendering them responsive to light stimuli and enabling precise temporal and spatial modulation of their activities, as mentioned in Figure 3 [73]. The delivery of light with specific wavelengths to targeted regions of the brain via tools such as fiber optics is a common practice in this field. Upon activation by light, opsins undergo a conformational change, thereby facilitating the movement of ions across the neuron’s membrane to either excite or inhibit its function [75]. This meticulous modulation of neuronal activity has encouraged researchers to explore the functions of distinct neurons or neural circuits in intricate brain processes and conditions such as epilepsy. Optogenetic procedures in epilepsy involve the use of light stimulation to study and manipulate neural circuits associated with seizures. The procedure can be used to promote seizures, either with light stimulation alone or in combination with electrical kindling, as shown in Figure 3.

In epilepsy models, optogenetics has been employed to abate seizures by activating tonic spiking in the thalamus or stimulating cerebellar neurons, demonstrating its effectiveness in modulating neural circuits associated with seizures. Optogenetics has also been utilized to induce seizures by stimulating specific cell types, such as ChR2‐expressing cells in the hippocampus, shedding light on the contributions of different cell populations to seizure activity. Optogenetics studies have shown that the manipulation of GABAergic interneurons can either increase or decrease seizure activity, highlighting the importance of inhibitory neurotransmission in modulating neuronal excitability and seizure generation [76].

Challenges in applying optogenetic intervention in human epilepsies include the need for powerful light sources to access deep brain structures and the requirement for accurate, real‐time seizure detection. Issues such as time‐consuming procedures for virus injection and ferrule implantation are being addressed with near‐infrared versions of ChR2 that do not need ferrule implants for light delivery.

### 6.2. Artificial Intelligence and Machine Learning

Deep learning has been extensively utilized in the automated processing of EEG data in various domains, such as brain–computer interfaces, automatic sleep scoring, and the prediction and detection of epileptic seizures, owing to its ability to extract intricate representations from unprocessed data [77]. This research focuses on the CHB‐MIT scalp EEG database, comprising EEG signals from 23 pediatric patients with intractable seizures at Boston Children’s Hospital, and the EPILEPSIAE project dataset, which includes high‐quality EEG recordings from 275 epilepsy patients, encompassing both scalp and intracranial recordings. The EPILEPSIAE dataset contains crucial metadata related to the recordings and patients, such as seizure onset and offset times, electrodes used, and additional seizure classifications, all of which are meticulously annotated by expert neurologists [78]. Deep learning techniques facilitate automated seizure detection by directly learning detailed representations from raw EEG data, thus eliminating the need for manual selection of features. Deep learning operates through neural networks with multiple layers to grasp complex patterns and representations from the data, enabling automatic extraction of features without human intervention. Through the employment of fully convolutional neural networks, deep learning has demonstrated remarkable accuracy and specificity in tasks such as seizure detection. These approaches do not rely on predefined features, hence decreasing the need for human involvement and enhancing efficiency in the process. By leveraging fully convolutional neural networks, exceptional accuracy and specificity levels are attained, such as 99.3% accuracy and 99.6% specificity for the CHB‐MIT dataset and 98.0% accuracy and 98.3% specificity for the EPILEPSIAE dataset. The incidence of false alarms per hour is notably low, with rates below 0.5/h for 92% of CHB‐MIT patients and below 1.0/h for 80% of EPILEPSIAE patients [79].

Several software tools are employed in the identification and examination of seizures, primarily through the analysis of EEG data. Persist is a notable platform that provides real‐time seizure detection, review of EEG data, and generation of reports, utilizing advanced algorithms to recognize seizure patterns and anomalies. Biosemi Analyzer offers similar functionalities, catering to both real‐time monitoring and offline analysis of EEG recordings. The BrainVision Analyser is another widely utilized software package that encompasses features for seizure detection, removal of artifacts, and spectral analysis. MATLAB, which is frequently utilized alongside the EEGLAB toolbox, is highly preferred in research settings because of its adaptability in creating bespoke algorithms for seizure detection based on EEG data.

### 6.3. Focused Ultrasound

Focused ultrasound is a cutting‐edge technology with diverse applications in various fields, including oncology, neurology, and neuromodulation. Research has shown that focused ultrasound can be utilized for non‐invasive targeted destruction of tumors, enhancing immune responses to cancer, and opening the blood–brain barrier for drug delivery in conditions such as Parkinson’s disease, Alzheimer’s disease, and epilepsy. Focused ultrasound has the potential to revolutionize treatments by offering precise interventions in brain circuits for neurological purposes.

**Figure 4:**
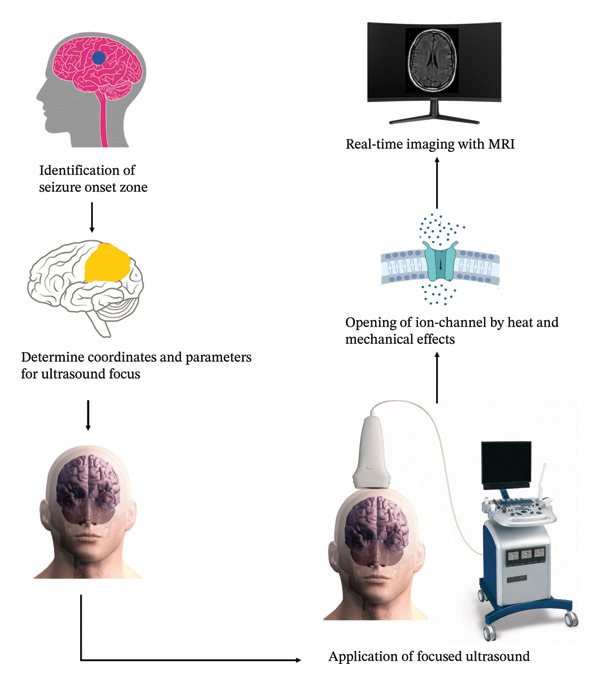
Illustration depicting a possible approach for treating epilepsy via focused ultrasound. It describes a procedure that commences by identifying the location of seizure initiation within the brain through real‐time MR imaging. The specific coordinates and parameters for directing the ultrasound waves are subsequently established based on these data. The patient’s head is then fixed in place, and a targeted application of focused ultrasound is administered. It is hypothesized that ultrasound facilitates the opening of ion channels within brain tissue via thermal and mechanical mechanisms, potentially interrupting the abnormal neural activity linked to seizures.

In focused ultrasound, one or multiple ultrasound beams of varying intensities, whether low or high, modulate cerebral activity or eliminate neuronal matter, respectively, characterizing this technique. These beams, which are composed of high‐pressure waves, originate from a pulse generator and are enhanced by a transducer [80]. By directing the beam toward a specific point within the brain, the result is the generation of acoustic energy precisely at the intended location, as shown in Figure 4. Integration of focused ultrasound with magnetic resonance imaging guidance is commonplace for precisely delineating the target tissue with millimeter accuracy and monitoring the effects of lesioning throughout the focused ultrasound procedure [81]. A standard sonication protocol involves the consideration of five key parameters: the fundamental frequency, pulse repetition frequency, duty cycle, duration of sonication, and intensity. Adjustment of these parameters can influence the characteristics, extent, and spatial selectivity of the impact [82].

High‐intensity focused ultrasound has obtained FDA approval for the management of essential tremor and Parkinson’s disease, in addition to various non‐neurological conditions. Focused ultrasound mechanically deforms mechanosensitive ion channels in cellular membranes, potentially leading to channel opening, ion influx, cell depolarization, and activation of voltage‐gated ion channels.

## 7. Practical Considerations for Emerging Neurotechnologies

Although developing neuromodulation and seizure monitoring devices shows potential, certain significant obstacles hinder their broad implementation. Some of the practical considerations are explained below.

### 7.1. Device Availability

Device availability is restricted due to limited regulatory clearances, country‐specific import limitations, and the concentration of clinical trials in specialized epilepsy facilities. Expenses can be exorbitant—implantable EEG systems (e.g., subscalp or responsive neurostimulation devices) may surpass $20,000–$40,000 for the device and implantation, not including follow‐up expenses, whereas sophisticated neuromodulatory techniques such as focused ultrasound or experimental optogenetics necessitate costly imaging, surgical, and hardware infrastructure [83, 84].

### 7.2. Insurance Coverage

Insurance coverage is highly variable; in the U.S., certain FDA‐approved devices, such as RNS and VNS, receive reimbursement for certain clinical purposes, while emerging technologies, such as subscalp EEG and wearable seizure‐detection devices, frequently lack uniform coverage policies. In numerous nations, reimbursement is either limited or nonexistent, leading to substantial out‐of‐pocket expenses [84].

### 7.3. Training Requirement

Training prerequisites constitute a significant obstacle. Technologies like focused ultrasound necessitate multidisciplinary teams proficient in neurosurgery, neuroimaging, and epilepsy management, whereas optogenetic methods require specialized surgical skills and gene delivery protocols that are presently confined to research environments. Wearable systems necessitate training for accurate placement, data interpretation, and interaction with clinical operations [84].

### 7.4. Maintenance and Follow‐Up

Moreover, the long‐term maintenance and follow‐up obligations might be considerable, particularly for implanted devices necessitating regular battery replacements, recalibrations, or hardware updates. Concerns regarding data privacy and cybersecurity are becoming increasingly pertinent for cloud‐connected wearable and implantable equipment, as breaches may threaten important patient health information [54].

## 8. Discussion

Technologies for managing epilepsy range from fully invasive systems, including intracranial EEG and implanted neurostimulators, to non‐invasive alternatives such as surface EEG and external stimulation methods. Invasive systems offer enhanced signal fidelity, better spatial resolution, and precise therapeutic effects; however, they involve increased procedural risks, higher costs, and limited accessibility. Non‐invasive methods prioritize safety, portability, and patient comfort; however, they may have limitations in detecting accuracy for focal seizure activity and in maintaining long‐term continuous monitoring [42]. Emerging intermediate approaches combine advantages from both ends of the spectrum, providing a practical middle ground for long‐term seizure management. Subscalp EEG systems, such as EpiMinder Minder and UNEEG 24/7 EEG SubQ, are minimally invasive devices placed beneath the scalp but outside the skull. These systems enable extended high‐quality recordings over months or years while reducing surgical risks compared to intracranial electrodes. They also reduce motion and impedance‐related artifacts associated with surface EEG and eliminate the need for craniotomy, making them more feasible for wider use. In‐ear EEG systems, currently under development, use discreet electrodes within the ear canal for continuous brain activity monitoring, improving patient compliance and social acceptability compared to traditional scalp electrodes. Though less invasive than subscalp implants, they may provide more stable signals than wearable headbands, potentially improving seizure detection in ambulatory settings. Targeted infusion systems, such as intraventricular pumps and CED catheters, are moderately invasive therapies that deliver drugs directly to epileptogenic zones, reducing systemic exposure linked to oral or intravenous treatments.

These systems can be implanted percutaneously, lowering morbidity relative to open surgery, and can be adjusted or removed as needed, supporting chronic management. The practical benefits of these intermediate approaches include higher patient acceptance than highly invasive procedures, better long‐term monitoring accuracy than purely non‐invasive devices, potential cost savings through fewer hospital admissions for uncontrolled seizures, and the ability to be removed or deactivated without causing permanent structural changes. Key factors before widespread adoption include long‐term biocompatibility, surgical training needs, device durability, and data infrastructure. Regulatory pathways for intermediate devices may differ from those for noninvasive wearables and traditional implants, requiring specific approval and reimbursement strategies. The development of intermediate systems indicates that their integration into epilepsy care will likely involve a hybrid model. This approach should tailor device selection based on seizure type, origin, patient comorbidities, and lifestyle preferences. Such a balanced approach can potentially improve both efficacy and tolerability, expanding access to advanced seizure monitoring and treatment for more patients.

Emerging intermediate approaches integrate selected advantages from both extremes of the spectrum, presenting a viable middle ground for the long‐term management of seizures. Subscalp EEG systems, including EpiMinder Minder and UNEEG 24/7 EEG SubQ, are minimally invasive devices positioned beneath the scalp yet external to the skull. These systems facilitate extended periods of high‐quality recording, ranging from months to years, while presenting a reduced surgical risk in comparison to intracranial electrodes. These systems mitigate various motion and impedance‐related artifacts associated with surface EEG and obviate the necessity for craniotomy, thereby enhancing their feasibility for broader application [38, 40].

In‐ear EEG systems, presently under development, utilize discreet electrode placement within the ear canal to facilitate continuous monitoring of brain activity, enhancing patient compliance and social acceptability relative to conventional scalp arrays. Although less invasive than subscalp implants, they may provide greater signal stability compared to wearable headbands, which could enhance seizure detection in ambulatory environments.

Targeted infusion systems, including intraventricular pumps and CED catheters, serve as a moderately invasive therapeutic approach. They facilitate the direct delivery of drugs to epileptogenic zones, thereby minimizing systemic exposure associated with oral or intravenous therapies. These systems can be implanted percutaneously, resulting in reduced morbidity compared to open resection, and they can be adjusted or removed as necessary, thus facilitating chronic management [63, 66].

The practical advantages of these intermediate approaches include improved patient acceptability relative to highly invasive procedures, enhanced long‐term monitoring accuracy compared to purely non‐invasive devices, potential cost‐effectiveness through decreased hospital admissions for uncontrolled seizures, and the ability to remove or deactivate without causing irreversible structural changes. Key considerations before large‐scale adoption include long‐term biocompatibility, surgical training requirements, device longevity, and data management infrastructure. Additionally, the regulatory pathways for intermediate devices may vary from those of non‐invasive wearable and traditional implants, requiring specific approval and reimbursement strategies [61, 83, 85].

The evolution of intermediate systems suggests that their integration into epilepsy care will necessitate a hybrid model. This model should personalize device selection according to seizure type, anatomical origin, patient comorbidities, and lifestyle preferences. This balanced approach has the potential to reconcile efficacy and tolerability, thereby enhancing the accessibility of advanced seizure monitoring and treatment for a wider patient demographic.

## 9. Conclusion

Epilepsy presents considerable challenges as a neurological condition characterized by recurrent seizures, demanding sophisticated medical devices for efficient management. The utilization of implantable devices has exhibited clinical effectiveness; however, they are linked with surgical risks and long‐term implications, underscoring the necessity for individualized therapeutic approaches. Devices, such as EEG‐based wearables and mobile applications, provide convenience and continuous monitoring capabilities, thereby enriching patient care. Prospective advancements in technologies such as focused ultrasound, optogenetics, and AI‐driven seizure prediction show promise but mandate thorough validation and the resolution of obstacles related to safety, accessibility, and clinical verification. Collaborative endeavors in research and clinical trials are imperative for the progression and integration of these technologies into all‐encompassing, personalized care strategies for individuals with epilepsy to enhance outcomes and improve quality of life.

## Author Contributions

Mukul Shyam: conceptualization, methodology, and writing–original draft preparation. Naincy Kaushal: reviewing and editing. Manish Yadav: reviewing and editing. Prathap Srirangan: writing–original draft preparation and image preparation. Sabina Evan Prince: visualization and supervision.

## Funding

No funds, grants, or other support were received.

## Ethics Statement

The authors have nothing to report.

## Conflicts of Interest

The authors declare no conflicts of interest.

## Data Availability

Data sharing does not apply to this article as no dataset is generated or analyzed during the current study.
